# Exploring the Feasibility of Appreciative Inquiry on Job Satisfaction of Intensive Care Nurses: A Mixed‐Method Study

**DOI:** 10.1155/jonm/9183130

**Published:** 2026-09-01

**Authors:** Wai Chun Ho, Chi Tim Hung, Lung Kwan Tang

**Affiliations:** ^1^ Intensive Care Unit, Queen Elizabeth Hospital, Hospital Authority, Hong Kong, China, qehconnect.com; ^2^ The Jockey Club School of Public Health and Primary Care, The Chinese University of Hong Kong, Hong Kong, China, cuhk.edu.hk

**Keywords:** appreciative inquiry, job satisfaction, strength-based

## Abstract

**Introduction:**

The increasing nursing attrition rate has become a major concern. Job satisfaction is a key predictor of nurse turnover, yet the effectiveness of strength‐based approaches remains underexplored. This study aimed to explore the feasibility and preliminary effects of implementing Appreciative Inquiry (AI) on job satisfaction among intensive care unit (ICU) nurses.

**Methods:**

Following ethical approval, AI using the 5‐D cycle was implemented over a five‐month period with ICU nurses. The intervention involved a series of sessions designed to explore the nurses’ best experiences, core values, and wishes through dialog and inquiry. The Minnesota Satisfaction Questionnaire (MSQ) was administered pre‐ and postintervention to assess job satisfaction changes, and focus group interviews were conducted to gather qualitative insights.

**Results:**

Sixty‐three ICU nurses participated in the study. Quantitative analysis showed no statistically significant increase in general job satisfaction (mean score: 68.8 to 70.0) and intrinsic satisfaction (43.4 to 44.2). However, specific MSQ subitems, particularly those related to receiving praise and sense of accomplishment, showed preliminary improvements (*p* < 0.05), although these findings are exploratory. Qualitative findings revealed that impactful nursing care often stems from personal interactions, highlighting why nurses find meaning and appreciation in their work. Many participants expressed interests in exploring more appreciative topics, suggesting AI’s potential for broader workplace improvement.

**Conclusion:**

AI is a novel approach within the Hong Kong nursing context. While overall findings did not reach statistical significance, this study suggests AI application within an ICU setting is feasible. The preliminary feedback indicates its potential to enhance staff engagement and targeted aspects of job satisfaction. Further controlled and rigorous research is recommended to explore the AI impact on healthcare environments.

**Implications for Nursing Management:**

Nurse managers can practically integrate AI by using strength‐based dialog and inquiry to focus on best practices during staff reviews, targeting specific workplace stressors, and implementing structured recognition practices to celebrate staff successes.

## 1. Introduction

Talent retention in nursing is critical for maintaining high‐quality patient care and sustainable healthcare development [1, 2]. The Hospital Authority has reported significant nursing attrition rates, reaching 12.8% in 2021/2022 [3], and the intensive care unit (ICU) faces similar challenges. Prior to initiating this study, informal exit interviews using structured questions were conducted with departing nurses to understand the proximal and distal factors behind their decisions. These interviews revealed that dissatisfaction primarily stemmed from increasing workloads and a lack of a positive, supportive atmosphere. Additionally, it has been observed that ICU nurses elsewhere often exhibited signs of low job satisfaction, including a lack of enthusiasm, negative attitudes, and escalating resignation rates. Local and international literature highlights that while a nurse’s job satisfaction is crucial for staff retention, workplace morale and patient care quality, it is negatively correlated with job stress and the intention to quit [4–6].

In the usual context of nursing and general healthcare, a common practice for addressing issues is adopting a deficit‐based approach, which often perpetuates negative feelings, stress, and resentment [7]. This focus on problem analysis frequently resulted in discouragement, draining energy from staff to change. Considering the negativity stemming from the current deficit‐based approach, a novel strength‐based approach, Appreciative Inquiry (AI), may serve as an alternative method for initiating positive culture changes in nursing and the broader healthcare sector.

### 1.1. AI and Job Satisfaction

Job satisfaction is a multifaceted concept referring to individuals’ subjective feelings and attitudes toward their jobs and various associated aspects [8]. According to Herzberg’s Two‐Factor Theory, the presence of intrinsic motivators (e.g., achievement, recognition, and growth) is a key driver of job satisfaction, while a lack of hygiene factors (salary, policies, and regulations) can lead to dissatisfaction [9]. The Need Fulfillment Theory [10] and Affective Event Theory [11] further emphasize the importance of meeting staff needs and building positive emotional experiences in the workplace, incorporating an understanding of the cognitive and emotional processes that contribute to job satisfaction. To counter the prevailing unsupportive environments and lack of positivity in healthcare settings, a strength‐based approach that uncovers distinctive positive events, personal growth, and achievements is hypothesized to significantly improve job satisfaction.

AI, featuring a 5‐D cycle, was developed in 1987 as an alternative to the traditional problem‐solving approach in organizational development [12]. The rationale behind AI is to focus on conversations that matter. In every society, certain elements work effectively, and our focus shapes these multiple realities. Individuals can be positively influenced through inquiries and dialogs, making them more confident in using best practices from the past to face current challenges [13]. The aspiration is that, through constructive collaborations, the working culture can transform into one that is more positive, generative, and strength‐based, thus improving job satisfaction through an alternative approach. However, little research has been conducted to explore the impact of AI on job satisfaction. The objective of this pilot study is to assess the feasibility of implementing AI within the ICU and to explore preliminary changes in nurses’ perceptions and job satisfaction.

## 2. Methods

### 2.1. Study Design

This study utilized an exploratory, mixed‐methods, single‐group pre‐ and postintervention design to evaluate the feasibility and preliminary impact of AI on job satisfaction among ICU nurses at a public hospital over a five‐month period (January to May 2024). The primary outcome was the change in general and intrinsic job satisfaction scores following the intervention. Nurse resignation rates within the department were also tracked to provide descriptive context regarding staff retention. Pre‐intervention baseline data (October 2022 to October 2023) and postintervention data (September 2024 and September 2025) were recorded to observe general staffing trends. A mixed‐method approach was chosen to allow quantitative data to capture broader trends in job satisfaction, while the qualitative components provided contextual data about nurses’ experiences. Institutional ethics approvals were obtained prior to the study. To enhance the quality of the AI intervention, the first author completed a certified training to ensure a comprehensive understanding of AI principles and interview techniques.

### 2.2. Participants and Sampling

A universal sampling of the eligible nursing staff within the department was employed as the quantitative baseline, yielding a participation rate of 72% (*n* = 63) of the available targets, which minimized selection bias as possible. Eligible participants were approached face to face by the first author during shift breaks and invited to participate voluntarily. Participants who consented to the study by signing a written consent form were engaged in structured AI processes. A physical information sheet was also provided to participants to explain the study design with confidentiality ensured throughout the study. In view of the overall ICU patient care and promoting AI to nursing staff, target participants were registered nurses (RNs) and advanced practice nurses (APNs) irrespective of their length of service. Nurses planning to resign or retire in the study period were excluded to ensure that participants could complete the intervention.

For the qualitative components, a purposive sampling of the baseline participants was recruited. Appreciative interviews were conducted with 36 nurses (30 RNs and 6 APNs). After the intervention, two focus group interviews were conducted and facilitated by the first author to gather qualitative feedback from a total of 9 participants: one group of 5 nurses with 1–3 years of clinical experience and a second group of 4 nurses with 5–11 years of clinical experience.

### 2.3. Data Collection

#### 2.3.1. Quantitative Data

The Minnesota Satisfaction Questionnaire (MSQ) short form was administered to collect baseline job satisfaction and demographic data, including gender, rank, and seniority (years of experience). This questionnaire, demonstrating good reliability and validity, was available in a mixed Chinese and English version to accommodate participants’ preferences. [14–16]. After collecting the pre‐intervention job satisfaction, AI processes were deployed as the intervention. The postintervention MSQ was distributed to measure any differences in job satisfaction.

#### 2.3.2. Qualitative Data

The first author conducted one‐on‐one appreciative interviews utilizing a structured and validated protocol (Table 1) without additional facilitator involvement. Interviews lasted approximately 5–10 min and were conducted during free time duties to minimize interference. Secondly, postintervention focus group interviews were conducted to gather qualitative feedback about the intervention so as to offer explanations of why some changes have occurred and recommendations for future workplace applications. Focus group interviews lasted approximately 30 min. All interview contents were recorded by field notes only. Data were expanded and transcribed immediately after the interview into word documents with anonymous number tags to maximize data quality [17].

**TABLE 1:** The structured and validated appreciative interview protocol for ICU settings.

Best experience:
1. I would like you to think back over your work experience as a nurse and recall a time when you were most engaged in a real and meaningful way. Tell me about that nursing care and why this particular experience stands out as a high point of engagement for you.
2. Reflecting on it, what strengths and behavior did you and others exhibit that enabled meaningful engagement?
3. What would you say are the necessary or key factors that make meaningful engagement possible?
*Values:*
Let’s talk for a moment about some things you value deeply; specifically, the things you value about yourself.
4. What do you value most about yourself as a person and as a member of this hospital?
5. What are you feeling most engaged and fulfilled in your work? What are you doing? What do you value most about this work?

*Wishes:*
Imagine it is one year from now and the ICU has achieved the effective outcomes, among others, and other hospitals want to understand how they can learn from you. A nurse from another hospital asks the questions.
6. What did ICU do to facilitate such high levels of engagement among doctors, nurses, and relatives?
7. What changes did you make to support engagement and reinforce new practice?
8. Anything you would like to share?

### 2.4. Application of AI Through a 5‐D Cycle (Table 2)

**FIGURE 1:**
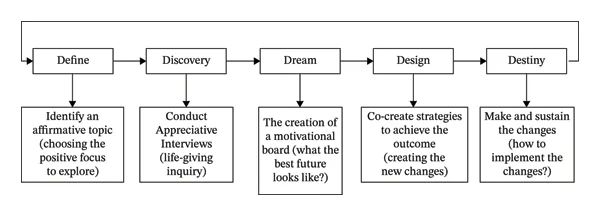
The illustration of a 5‐D cycle of Appreciative Inquiry [18].

AI acts as an intervention to create a paradigm shift through a 5‐D cycle with 5 interconnected phases: Definition, Discovery, Dream, Design, and Destiny [18] (Figure 1).

**TABLE 2:** A summary table of the 5‐D Appreciative Inquiry application.

	Core activities	Frequency	Participants
1. Define	Established the affirmative topic from reframing the problems as the focus of the inquiry	Once	Nurse Consultant
2. Discovery	Conducted interviews to identify positive themes	One‐time sessions with each participant	Nursing staff
3. Dream	Highlighted positive themes and celebrated some “small victories” from interviews to other staff	Ongoing	Nursing staff
4. Design	Presented the Discovery findings with possible strategies (promoting appreciative culture etc.)	Once	Managers
5. Destiny	Incorporated appreciative practices into daily routine and track the changes and responses	Ongoing	Nursing staff

#### 2.4.1. Definition

This first step involved reframing problems into affirmative topics through dialogs. An example is reframing the “causes of patient harm” into “excellent patient safety.” The goal of this stage is to reach agreement on the scope of the study. After a thorough discussion with an ICU Nurse Consultant “Impactful nursing care” was identified as the affirmative topic.

#### 2.4.2. Discovery (Appreciative Interviews)

During this phase, one‐on‐one interviews were conducted with nurses to explore their best experiences, values, and wishes. A structured interview protocol (Table 1) tailored to the ICU settings was developed and validated by the Chief Operating Officer of the Centre for Appreciative Inquiry to ensure conceptual alignment and guide the conversation, ensuring consistency and quality of data collection across interviews. Data collection continued until data saturation was reached, defined as the point at which no new themes or core values regarding impactful nursing care emerged.

#### 2.4.3. Dream (Motivational Board)

Participants can amplify the positive aspects of the system by relating to the future. Based on the themes identified in the discovery phase, participants were asked about the desired future reality (i.e., “what will it be looked like?”). A motivational board was created by colleagues to share daily life photos. This unleashes the imaginary power to expand the system’s potential and shift to the desirable status quo.

#### 2.4.4. Design

Findings from the Discovery were presented to nursing managers to promote positive changes in the workplace. Based on the results, recommendations included promoting a culture of appreciation within the department, offering more diverse simulated trainings to enhance nurses’ skills, competency, and confidence, and conducting AI on other pertinent topics.

#### 2.4.5. Destiny

This phase focused on sustaining positive changes by building momentum to continue exploring and discussing the positive aspects in the workplace and incorporating appreciative practices into daily routines. The integration of AI principles and colleagues’ strengths was discussed with managers about broader shifts in departmental strategies.

### 2.5. Data Analysis

#### 2.5.1. Quantitative Analysis

The primary outcome was the change in general satisfaction scores, including intrinsic scores, which were analyzed using the paired *t*‐test by the Statistical Product and Service Solutions (SPSS) Version 27. The assumptions for the paired *t*‐test were verified prior to the testing. The difference of the two sets of satisfaction mean scores (pre‐ and postintervention) was tested for normality. A skewness of 0.42 (standard error [SE] = 0.30) and a kurtosis value (excess) of 0.64 (SE = 0.60) indicated that the data distribution was slightly positively skewed but contained no extreme outliers. The Shapiro–Wilk test also confirmed the data followed a normal distribution (*W* = 0.97, *p* = 0.27). Resignation rates were calculated using simple descriptive statistics to indicate the trends by percentage. A one‐year observation window (2022 to 2023 and 2024 to 2025) was chosen to provide sufficient time to observe the trends [19].

#### 2.5.2. Qualitative Analysis

Qualitative transcripts from both appreciative and focus group interviews were imported into NVivo software Version 15 for thematic analysis. To ensure analytic rigor and minimize individual bias, two authors (Wai Chun Ho and Lung Kwan Tang) first read the transcripts multiple times before analysis to enhance familiarization with the data and then coded the data independently. Each interview question and responding answer was carefully reviewed in order to build connections and understand the logic behind them. Created codes were systematically reviewed for recurring patterns, similarities, and differences to group them into respective themes. The themes were categorized regarding their features and natures in an inductive approach. After reviewing the 36 interview contents, no new subthemes or themes were generated, therefore defining that the data analysis reached a saturation point. The authors discussed and refined the subthemes and themes to ensure accurate representation of participants’ responses by constantly returning to the original transcripts to address divergence and ensure confirmability. The authors also continuously acknowledged their own role as ICU staff and consciously bracketed their preconceptions toward the interpretations during coding to address reflexivity.

#### 2.5.3. Mixed‐Methods Integration

Finally, a triangulation protocol was utilized to integrate quantitative and qualitative data. Qualitative themes derived from the appreciative and focus group interviews were mapped directly against specific MSQ subitem score changes (“Feeling of accomplishment” and “receiving praise”). This allowed qualitative narratives to be an explanatory mechanism for why specific aspects of intrinsic satisfaction showed preliminary changes, while general satisfaction remained unchanged.

## 3. Results

Sixty‐three ICU nurses participated in the study, with 47 RNs and 16 APNs (Table 3).

**TABLE 3:** Demographic characteristics of all participants (*n* = 63)^∗^.

Demographic characteristics	All participants (*n* = 63)
Gender	
Male	19 (30.2%)
Female	44 (69.8%)
Rank	
RN	47 (74.6%)
APN	16 (25.4%)
Seniority	
< 1 year	10 (15.9%)
1–5 years	15 (23.8%)
6–10 years	12 (19.0%)
11–20 years	12 (19.0%)
≥ 21 years	14 (22.2%)

Abbreviations: APN, advanced practice nurse; RN, registered nurse.

^∗^Data are shown as no (%).

### 3.1. The Satisfaction Scores

The mean general satisfaction scores and intrinsic satisfaction scores (Table 4) increased slightly, but these changes did not reach statistical significance. Among RNs, there was a slight increase in both general and intrinsic satisfaction, but changes were not statistically significant. In contrast, APNs experienced a decrease in both general and intrinsic satisfaction over the study period, with scores declining with no statistically significant difference (Table 4).

**TABLE 4:** Job satisfaction scores (mean) of the MSQ^∗^.

	Before AI	After AI	Effect size (ES) with 95% confidence interval (CI)	*p* value^†^
*General satisfaction scores (mean) (out of 100)*				
Overall	68.8 ± 8.6	70.0 ± 9.9	0.097 (−0.15 to 0.34)	0.44
RN	68.6 ± 7.9	70.7 ± 10.7	0.14 (−0.15 to 0.43)	0.35
APN	69.4 ± 10.5	67.9 ± 6.9	0.21 (−0.70 to 0.30)	0.42

*Intrinsic satisfaction scores (mean) (out of 60)*				
Overall	43.4 ± 4.8	44.2 ± 5.6	0.11 (−0.13 to 0.36)	0.37
RN	43.3 ± 4.4	44.7 ± 5.9	0.18 (‐0.11 to 0.47)	0.21
APN	43.8 ± 6.0	42.6 ± 4.5	0.24 (−0.72 to 0.25)	0.35

*Note:* A two‐tailed *p* < 0.05 was considered statistically significant.

Abbreviation: AI, Appreciative Inquiry; APN, advanced practice nurse; MSQ, Minnesota Satisfaction Questionnaire; RN, registered nurse.

^∗^Data are shown as mean ± standard deviation.

^†^Paired *t*‐test: *p* values were unadjusted for multiple comparisons.

Among the MSQ subitems, two statistically significant results were found (Table 5).

1. “The feeling of accomplishment I get from the job” with the score increased from 2.89 to 3.27 (*p* < 0.05).
2. “The praise I get for doing a good job” with the score increased from 3.38 to 3.65 (*p* < 0.05).

**TABLE 5:** Job satisfaction scores (mean) of the MSQ subitems^∗^.

	Before AI	After AI	ES with 95% CI	*p* value^†^
The satisfaction scores (mean) of subitems (out of 5)				
The praise I get for doing a good job	2.89 ± 0.76	3.27 ± 1.04	0.32 (0.57 to 0.067)	0.01
The feeling of accomplishment I get from the job	3.38 ± 0.79	3.65 ± 0.77	0.27 (0.52 to 0.021)	0.03

*Note:* *p* values were unadjusted for multiple comparisons. A two‐tailed *p* < 0.05 was considered statistically significant.

Abbreviation: AI, Appreciative Inquiry; MSQ, Minnesota Satisfaction Questionnaire.

^∗^Data are shown as mean ± standard deviation.

^†^Paired t‐test.

These results suggest a limited improvement on job satisfaction, but specific aspects of intrinsic satisfaction, such as receiving praise and experiencing a sense of accomplishment, showed positive preliminary changes following the AI intervention. However, as the *p*‐values were unadjusted for multiple comparisons, these two findings (“praise” and “accomplishment”) are exploratory.

### 3.2. Nurse Resignation Rates

It was observed that the overall nursing resignation rate was lower during the second timeframe (September 2024–September 2025) at 5.2%, compared to the initial period at 13.2% (October 2022–October 2023) (Table 6).

**TABLE 6:** Resignation rate between 2022 and 2025^∗^.

	October 22–23	September 24–25
Rank		
Overall	12 (13.2%)	5 (5.2%)
RN	10	5
APN	2	0

Abbreviations: APN, advanced practice nurse; RN, registered nurse.

^∗^Data are shown as no (%).

### 3.3. Appreciative Interview Summary

A total of 36 interviews were conducted in the Discovery phase (57.1% of participants, 30 RNs and 6 APNs) over a two‐month period. Though not a verbatim transcript, participants’ responses were captured as accurately as possible and organized into themes.

There were four themes identified regarding the impactful nursing care:

Theme 1: Communication and rapport building: Relationship building with patients, relatives, and colleagues is important and creates sense of recognition.

“I introduced myself, and I’m happy that the patient remembered my name. It′s rare for patients to recognize staff, even when conscious.”

“I believe attitude matters. While it can be difficult to change someone’s usual practice, positive influence does happen when I continue doing the right things.”

Theme 2: Emotional and psychological support: Providing these supports to patients and relatives is fulfilling the basic nursing role.

“In a past project, I encouraged relatives to give physical massages to critically ill patients. Many appreciated the opportunity to actively support their loved ones, rather than just standing by.”

Theme 3: Handling end‐of‐life issues: Provision of advanced nursing care brings satisfaction and fulfillment.

“I used a technique called the “Four Themes of Life—Gratitude, Apology, Love, and Farewell” [道謝、道歉、道愛、道別] to help relatives go through the final journey of a dying patient. It was a relief to them but also an achievement to me that they peacefully say thank you and goodbye to their father”

“Back to COVID pandemic, I was a case nurse of a dying patient. All visitors to ward were prohibited except close relatives. Relatives requested for a Baptism which was impossible to perform at that time. Coincidently I am a Christian, and I offered I could pray for the patient. The patient died soon after I prayed and the relatives showed much gratitude and even now I still keep contact with them.”

Theme 4: Maintaining patient dignity and well‐being: Performing simple care for patients helps maintain patient dignity and well‐being, imparting a sense of meaning to the profession.

“I remembered that I helped a patient shave, and he felt genuinely cared for, which left him with a lasting sense of being taken care of.”

“I was impressed and moved by the morning routine of oral hygiene and shaving. It helps re‐gain patients’ dignity and self‐image after tidying their appearance properly.”

The core components regarding nursing care (core values) were organized into three themes:

1. Nursing professionalism and clinical skills.
2. Recognition, motivation, and professional identity.
3. Supportive culture and team dynamics.

There were three themes of approach to improvement (wishes):

1. Attitude and behavioral enhancement.
2. Build a supportive and appreciative culture.
3. Protocol, training, and guidelines enhancement.

These narratives underscored nurses’ best experiences in their professional careers and interpersonal practices to achieve effective outcomes and indicated the complexity of nursing work and the intrinsic motivators that drive job satisfaction, such as recognition, teamwork, and making a difference in end‐of‐life care to patients and their families. The stories are moving and deserved to be made known to others for learning and sharing.

The interview contents (see supporting table 1) also revealed that ICU nurses gained a sense of achievement and satisfaction when being recognized and appreciated for nursing professionalism and clinical skills, which include communication, knowledge and application of nursing care, and meticulous wound management. Additionally, nurses value a supportive culture and positive team dynamics in the ICU, where rendering support among colleagues is crucial, especially during critical moments such as resuscitation and demanding procedures.

### 3.4. Focused Group Interview

The self‐reflection process helped nurses appreciate the positive impacts of patient care by answering thought‐provoking questions. They commonly shared that storytelling about personal peak experiences provided them with acknowledgment and appreciation. Interviews also uncovered positive indicators of team processes, such as improved engagement in communication and the reinforced belief that their behavior positively influences others. Participants recognized that cultural changes require broader involvement from leadership and management.

“I like this kind of interview but the topics can be more diverse.”

The interviews also highlighted that some senior nurses found it difficult to link positive stories to their overall job satisfaction.

“It is difficult to think of some positive events at first, but the questions helped me reflect on the value of being a nurse.”

Instead, they emphasized the workload and stress as sources of dissatisfaction, and what they appreciated and found more satisfying was the support and understanding from their colleagues and managers, such as nonjudgmental gestures, mutual trust, and receiving appreciation for solving problems. Conversely, they noted that the practice of criticizing poor performance negatively affects job satisfaction.

“It is difficult to find satisfaction through appreciating myself through this topic. In fact, less judgment is just fine to me.”

Some participants, however, expressed an interest in exploring other AI topics, such as knowledge, skills, and patient care pathways.

## 4. Discussion

This study provides preliminary insights into the feasibility of utilizing AI to address workplace culture and job satisfaction among ICU nurses. While the single‐group design precludes establishing a causal relationship between AI and job satisfaction, the mixed‐method approach provides a valuable exploration of a strength‐based intervention in a clinical environment and how colleagues respond to it. It offers a simple strategy to introduce positivity into a stressful work environment by relating colleagues’ past best experiences to their present feelings and future aspirations. Grounded in research and theories, AI is a complex process to influence one’s beliefs, behaviors, and motives. Bushe [20] stated that focusing on generative thinking could help build an appreciative mindset and enhance individual positive feelings. The qualitative findings complemented this interpretation, suggesting that staff may have enhanced their sense of accomplishment by actively recalling the best experiences and receiving appreciation through dialogic inquiries. Isen [21] also indicates that positive affect creates flexibility in thinking, leading to improved decision‐making, problem‐solving, and coping skills. AI introduces a fresh approach to invite nurses to verbalize their successful stories. The processes prompted nurses to reflect on their achievements, evoking positive emotions that inspired meaningful changes in their perspectives and actions.

The Discovery phase, which focused on uncovering personal accomplishments and peak experiences, helped nurses reconnect with motivation. The subsequent Dream, Design, and Destiny phases involve co‐creating and implementing strategies to drive transformative change. Neuroscience, such as mirror neurons, the placebo effect, and the Pygmalion effect, suggests that positive interactions and expectations may synchronize emotions and behaviors with a contagious effect, shaping a more positive future [13, 22].

Unconditional positive questions and storytelling reframed nurses’ perspectives and helped them focus on their own valuable experiences and beliefs. These specific questions touched on peak experiences, exploring not just what happened but also how and why it happened and personal values that contributed to those successes. One nurse even became tearful after sharing the story of a dying patient.

“It was a dying and relatively young patient receiving venoarterial extracorporeal membrane oxygenation (ECMO). I was first questioning myself … He should not be starting ECMO. It just prolonged his suffering … Until his wife and other relatives came … the artificial vital signs helped them gradually accept the situation and allowed doctors and I to explain the situation to them. Finally, I accompanied his wife to say goodbye to the patient and we switched off the ECMO.”

These “life‐giving” questions guided the nurse to explore a deeper sense of purpose and values when caring for the bereaved, moving beyond external praising to tap into intrinsic motivation and foster more lasting impact [13]. Although stimulating high‐quality motivation, such as internalized or intrinsic motivation, is crucial, external recognition and appreciation also support creating an appreciative atmosphere [23]. More appreciative actions were observed during the study, such as praising good nursing care and expressing appreciation toward staff in daily communications. Bushe [23] further distinguishes between “praising” and “blessing” in his earlier work, describing that praise involves recognizing and valuing something already completed, while a blessing truly appreciates the impact one is currently making. Cultivating a positive working environment with both praising and blessing is essential within a steep hierarchical structure. As more leaders in the workplace adopt AI principles, the creation of positive energy is likely to emerge, driving systemic change [20]. In line with this, management began implementing corresponding actions such as offering more simulated trainings to build nurses’ competence and strengthen their skillsets.

Notably, negativity bias initially made participants hesitant to identify strengths, but once successful stories were shared, nurses became more at ease responding to strength‐focused questions. Best practices and small victories deserve more attention. Leadership in AI is fundamentally about “choosing to focus.” If the focus is placed on mistakes, management style becomes deficit‐based. If the focus shifts to strengths, many creative ideas aligned with those strengths can emerge to help overcome systemic weaknesses [22]. This approach is not abandoning the traditional method to solely focus on strengths. Cooperrider [22] recommends adopting the 80/20 rule, focusing 80% on strengths and 20% on areas for improvement, in order to achieve the “positivity ratio.” More specifically, strength, opportunity, aspiration, and valued results are the four areas to be focused as the 80% strengths. Major transformations are often associated with one highly visible individual, and the management team is the key driver in reshaping the work atmosphere by leveraging various strengths and adopting AI principles.

In current healthcare settings, external regulations play a predominant role as a form of extrinsic motivation, standardizing practices to avoid undesirable consequences to safeguard the quality and safety of patient care. Negative phenomena, such as complaints and dissatisfaction, which arise both horizontally (among colleagues) and vertically (between management and frontline staff) have been observed when these standard practices are not met, often undermining intrinsic motivations. However, approaches to improvement suggested by colleagues may produce novel ideas to cater to staff needs. AI has the potential to inspire healthcare professionals to excel in their practice with renewed enthusiasm and commitment to achieving exceptional outcomes.

Another notable finding was the nonsignificant decline in job satisfaction scores among APNs. APNs bear a dual burden from clinical and administrative works. Qualitative feedback indicated that while the AI intervention was positive, it may not have sufficiently addressed the external stressors and heavy workload associated with APN roles. Senior staff indicated that while they valued appreciation, their satisfaction is heavily dependent on organizational support and workload management, which a short‐term and culture‐based intervention could not fully alleviate.

Meanwhile, the drop in resignations cannot be causally linked to the AI intervention because the study lacks a control group to account for broader workforce dynamics.

### 4.1. Challenges and Limitations

Limited time, noise, a lack of privacy, and brief interviews (5 to 10 min instead of the recommended 30 to 60 min) constrained deeper reflection [24]. This short duration was a compromise to minimize interference with patient care and ensure the highest possible participation rate within the busy work schedule among nurses. The short 5‐month study period may also have limited observable impacts, as some changes require years to fully manifest [25]. In addition, the chosen affirmative topic did not align with staff concerns, as some senior nurses felt it lacked relevance to their specific motivation. A more holistic exploration of workplace culture, including extrinsic satisfaction and staff needs, could have better flavored topic selection and outcomes.

There are several limitations to acknowledge. First, the single group design without a control group limits the ability to definitively attribute changes solely to AI interventions. Potential confounders include temporal effects, staffing fluctuations, leadership changes, and seasonal workload variation. Second, the generalizability of these findings is limited. This study was conducted in a single ICU within the public hospital system of Hong Kong. The unique culture, hierarchical structure, management style, and specific organizational stressors of this environment indicate that the results may not be directly applicable to other departments, private healthcare settings, or other regional contexts, influencing how nurses respond to strength‐based interventions compared to nurses in more decentralized healthcare systems.

Also, there is a vulnerability to Type I error for the *p* values which were unadjusted for multiple comparisons. The two significant findings (“praise” and “accomplishment”) are exploratory in nature. The sample size was also relatively small, limiting the statistical power to detect smaller effect sizes. Additionally, the first author acted as the primary interviewer. Although this ensured consistent application of AI principles, it may pose a confirmation bias and selection bias that affects data quality. In order to mitigate it, a second author was engaged in reviewing and coding transcripts independently to ensure the accuracy of participants’ views. This study solely relies on field notes to record interview data instead of verbatim transcripts and thus carries several limitations, including possible data loss, a high degree of subjectivity and research bias, and interference with the environment.

## 5. Conclusion

This study highlights the values of AI as a transformative approach but also reveals the complexities involved in its successful implementation. The storytelling of AI to collect best practices is a powerful tool and is a soft approach. The positive framing process elicited meaningful feedback, which could enhance the awareness of a positive‐based approach in handling problems. Further controlled and multicenter research is recommended for various aspects of impacts from AI.

### 5.1. Implications for Nursing Management

The findings offer practical strategies for nurse managers to integrate AI into daily leadership practices. Managers should consider incorporating appreciative questions into regular staff development reviews by means of strength‐based storytelling to encourage staff to share best practices and reflect on their workplace successes. Targeted interventions could be focusing AI on specific workplace concerns such as workload and organizational stressors. It is also important to create some moments of recognition and appreciation with structured recognition practices to highlight staff strengths.

## Author Contributions

Chi Tim Hung and Wai Chun Ho conceptualized the study design. Wai Chun Ho acquired the data. Wai Chun Ho and Lung Kwan Tang performed data analysis. Wai Chun Ho drafted the manuscript, and Chi Tim Hung and Wai Chun Ho provided critical revision for important intellectual content.

## Funding

This research received no specific grant from any funding agency in the public, commercial, or not‐for‐profit sectors.

## Ethics Statement

This study was reviewed and approved by the Research Ethics Committee (Kowloon Central/Kowloon East) of the Hospital Authority (Ref. no. KC/KE‐23‐0162/ER‐4) and the Survey and Behavioural Research Ethics Sub‐committee of Medicine of the Chinese University of Hong Kong (Ref. no. 023‐23). All participants provided signed consent before the study.

## Conflicts of Interest

The authors declare no conflicts of interest.

## Supporting Information

Additional supporting information can be found online in the Supporting Information section.

## Supporting information


**Supporting Information** The attached supporting table is a summary of appreciative interviews from thematic analysis with codes and themes identified.

## Data Availability

The data that support the findings of this study are available from the corresponding author upon reasonable request. The data are not publicly available due to privacy or ethical restrictions.
