# Supplementary material for: Exploring the Feasibility of Appreciative Inquiry on Job Satisfaction of Intensive Care Nurses: A Mixed‐Method Study
Source: J Nurs Manag. 2026 Sep 1;2026:9183130. doi: 10.1155/jonm/9183130 (PMC13531693; doi:10.1155/jonm/9183130)
Supplement: Supplementary file 1 [file JONM-2026-9183130-s001.docx]

Supplementary table 1. A summary of Appreciative interviews from thematic analysis

| Theme and code identified | Quote reference |
| --- | --- |
| Impactful nursing care | |
| Communication and Rapport Building | *“He (Patient’s son) became tearful and expressed relief, saying he felt much better after our conversation.”*  *“I was once praised by a patient for being empathetic as I spent some time staying and talking with him.”* |
| Emotional and psychological support | *“I regularly communicated with patients to check in on their needs and offered psychological support when they were sitting out of bed.”*  *“I provided psychological support for patients and their families. They thanked me for what I did to help them feel relieved.”* |
| Handling end-of-life issues | *“It was a dying and relatively young patient receiving venoarterial extracorporeal membrane oxygenation (ECMO). I was first questioning myself and my job whether I was doing it incorrectly. He was dying and should not be starting ECMO. It just prolonged his suffering and could not reverse his life. Until his wife and other relatives came, I started to change my perception. They cried so hard, especially his wife. However, the artificial vital signs helped them to gradually accept the situation and allowed doctors and I to explain the situation to them. Finally, I accompanied his wife to say goodbye to the patient and we switched off the ECMO.”*  *“It was heartbreaking to witness a loss at such an early age, especially when he [the patient] was the breadwinner of the family. I remembered a mother with two children visiting her husband at the bedside, crying and yelling. She insisted on not leaving the hospital even at midnight, saying, “what if he wakes up and cannot see me?” I told her to take a shower, have dinner and take her children home first and return to the hospital when she was more refreshed. She finally left the ICU.”* |
| Patient dignity and well-being | *“I helped a patient sit out of bed and it was rewarding to see their improvement, both mentally and physically, even though they were a bit tired during the process. They expressed appreciation for my support. It seems I made a difference in their recovery, which reminded me that I help them in the journey of the hospital stay.”*  *“[The interviewee] promotes patients’ dignity by removing the sputum stinks and stubble, bad breath and having a clean face again during hospitalization.”* |
| Nursing professionalism and clinical skills | |
| Knowledge and Application | *“A senior nurse taught me how to create a donut-shape pillow with foam for the head and neck support for a patient. I think it is a fine design which is creative and helpful, especially for patients with head wounds.”*  *“I managed a complicated surgical wound and had difficulty applying materials because the wound kept oozing. A senior nurse shared their experience and told me to use Duoderm Thin at the edge of the wound for grip first, and then apply Tegederm to cover the wound. The outcome was excellent. I could still observe the wound through the transparent dressing, while the Duoderm provided areas for re-applying other dressing materials.”* |
| Nurse roles | *“[The interviewee] identified the patient’s problem and inform doctors immediately if any issues occurred to ensure timely treatment.”*  *“[The interviewee reflected] The core value of nursing lies in providing care that helps reduce the physical and mental discomfort patients experience during their hospital stay.”* |
| Wound management | *“Incontinence associated dermatitis healed quickly when applied with appropriate barriers; that one I used was Cavilon spray.”*  *“I helped performed a wound dressing for a patient with a sternotomy wound. I could see the heart pumping at the first time and I had no ideas how to start the wound dressing. Eventually, a senior nurse taught me how to dress it in detail, and I still remembered it.”* |
| Recognition, Motivation and Professional Identity | |
| Achievement and Satisfaction | *“It is surprising and brings a great sense of achievement to see a patient’s progress when I applied these measures to them.”*  *“It is rewarding to see that some patients can be extubated and discharged from the ICU, because not everyone is able to fully recover and some remain dependent on ventilator or long-term care.”*  *“A critically ill patient with SLE and pulmonary hemorrhage was supported by ECMO. She remarkably made a full recovery and, after discharge from the ICU, occasionally visited us with gifts to express her gratitude. It was truly a miracle to me.”* |
| Recognition and Appreciation | *“They [relatives] thanked me for what I did.”*  *“Having hard work appreciated and recognized by colleagues afterwards.”*  *“Being told by others [other colleagues] that I was doing a good job with patients and relatives and receiving credit from them for doing so.”* |
| Supportive Culture and Team Dynamics | |
| Teamwork | *“We worked as a team and showed that nurses are capable of handling ACLS.”*  *“I appreciated our teamwork when handling a resuscitation of an expat. The process was smooth and swift and finally return of spontaneous circulation was achieved in a short period of time.”*  *“Team spirit in caring for patients in critical conditions.”* |
| Supportive environment | *“I remember during the SARS, staff were very supportive to each other. A culture of altruism was shown when colleagues purchased equipment out of pocket for other colleagues.”*  *It is my first time to dress a complicated wound. One has to be mentored to perform such complicated dressing.”*  *“I managed a complicated surgical wound and had difficulty applying materials because the wound kept oozing. A senior nurse shared their experience and told me to use Duoderm Thin at the edge of the wound for grip first, and then apply Tegederm to cover the wound. The outcome was excellent. I could still observe the wound through the transparent dressing, while the Duoderm provided areas for re-applying other dressing materials.”* |
| Approach to improvement |  |
| Attitude and behavioural enhancement | *“Casual mentioning your good work in conversation is a great way to praise what you did.”*  *“Providing more explanation and orientation to them could build the rapport.”*  *“Do not project a built-in profession status to patients or relatives that makes us distant from them.”*  *“Maybe someone else could see it and be influenced by such acts.”* |
| Build a supportive and appreciative culture | *“It is important to build a culture of physical and psychological care. The support of colleagues also plays a big role in providing care that goes beyond the standard.”*  *“Sharing information and knowledge with others is good practice”* |
| Protocols, guidelines, training enhancement | *“Some palliative care sharing or training could help staff face challenging situations and make them more at east when handling the problem or question raised by relatives.”*  *“Becoming familiar with the procedures, positions and roles”*  *“More simulated trainings sessions”* |
